# Clinical Profile of Patients Presenting With Eclampsia at a Semi-urban Tertiary Care Center

**DOI:** 10.7759/cureus.65651

**Published:** 2024-07-29

**Authors:** Rumi Bhattacharya, Milankumar Chaudhary, Shibashish Bhattacharjee, Romil kukadiya, Sheetal Shahu, Dipal Shah, Mamta R Patel

**Affiliations:** 1 Obstetrics and Gynecology, Pramukhswami Medical College, Bhaikaka University, Karamsad, IND; 2 Neurological Surgery, Pramukhswami Medical College, Bhaikaka University, Karamsad, IND; 3 Central Research Services, Pramukhswami Medical College, Bhaikaka University, Karamsad, IND

**Keywords:** magnesium sulfate, labetalol, perinatal mortality, maternal mortality, pre-eclampsia, eclampsia

## Abstract

Introduction

Pregnancies complicated by hypertensive disorders contribute to enormous burden on economy and health-care facilities. Eclampsia is one of the clinical markers of near-miss mortality. To achieve optimal outcomes, efforts should be directed at both periphery and tertiary care levels. This study aimed to compare the feto-maternal outcome in patients presenting with eclampsia and a matched control population.

Methodology

A comparative observational study was conducted among 70 cases and 70 controls. Detailed history and general and obstetrical examinations were carried out. Data was extracted from case files, labor room, and ICU records. Maternal and fetal outcomes were noted from January 2023 to January 2024. Statistical software STATA 14.2 (StataCorp LLC, College Station, Texas, USA) was used for data analysis. Observational descriptive statistics and chi-square and Fisher extract tests were applied.

Results

In our study, the incidence of eclampsia was 0.7% (70 per 1000 live births). The maternal mortality rate was 102.8/100000 live births and the perinatal mortality rate was 10.2/ 1000 live births in our study. The study observed a relatively young aged population and a significant bulk of cases belonged to late gestation or post-partum. Events like HELLP syndrome, abruption, liver, and renal failure were found to be frequently linked to eclampsia. Neonatal asphyxia (P-0.005), NICU requirement 41.43% vs 29% (P<0.01) preterm delivery 45.7% vs 14% (P=<0.001), and low birth weight were more commonly observed among the cases than the controls.

Conclusions

Eclampsia was found to be a significant contributor to elevated rates of morbidity and mortality in mothers and newborns. Poor antenatal care, severe anemia, and late referrals were some of the modifiable risk factors. Health care and economic burden on society is immense due to the significant utilization of intensive care and high dependency units.

## Introduction

Maternal morbidity and mortality are significant markers of healthcare utilization. Among different contributing factors, pre-eclampsia and eclampsia are only second to post-partum hemorrhage (PPH). Worldwide, it is estimated that this illness is responsible for 70,000 maternal fatalities [1]. Eclampsia is characterized by new-onset grand mal seizure activity and/or unexplained coma during pregnancy or postpartum in a female with signs and symptoms of pre-eclampsia. Descriptions of eclamptic seizures date back to 4000 years in ancient Greek, Chinese, and Indian literature. An idea of imbalance in the four humors was postulated as the genesis of this condition, and the term “Disease of theories” was coined for the same. Hippocrates was among the first to notice a preponderance of convulsions in first-time mothers [2]. As compared to developed countries, the burden of pre-eclampsia and eclampsia is conspicuously high in developing countries. The scenario in India is also quite alarming. At the Millennium Development Goals Summit (2015), world leaders pledged to reduce maternal and perinatal mortality rates [3]. Since then, several reformative programs have been introduced both at global and national levels to achieve the target [4,5]. The MMR in India has fallen to 99/100000 live births [6] and the perinatal mortality rate is 25.7/1000 live births [7].

According to the WHO report, the incidence of pre-eclampsia is seven times higher in developing countries. Similarly, the likelihood of progression to eclampsia is three times greater in developing countries (2.3 versus 0.8%) compared to developed countries [8]. In low- and middle-income countries, pre-eclampsia and eclampsia are responsible for 16% of all maternal deaths, according to the WHO [9]. Pre-eclampsia is a multisystem illness associated with dysfunction of the vascular endothelium, which results in multiple organ failure. Hypertension is usually the predominant finding in eclampsia. However, pre-eclampsia is often insidious in onset and evades most of the predictions and screening tests of detection. More importantly, hypertension may be absent in 16% of the cases, while proteinuria may be absent in 14% of the cases [10].

There is a large variance in the incidence of eclampsia in different parts of the world. It fluctuates between 0 and 0.1% in Europe, 0.6% and 9% in Brazil, 0.18% and 4.6% in India, and 9% in Nigeria [11]. Over 70,000 maternal deaths and over 5,000 neonatal and fetal deaths worldwide are attributed to pre-eclampsia and eclampsia each year [12]. Most maternal deaths in eclampsia occur due to severe complications like intracranial hemorrhage, pulmonary edema, hemolysis, elevated liver enzymes, low platelets (HELLP), abruption, disseminated intravascular coagulopathy, acute renal failure, hepatic failure or hemorrhage, adult respiratory distress syndrome, arrhythmias, etc. Intrauterine fetal growth restriction due to severe uteroplacental insufficiency, extreme prematurity, low birth weight, and fetal hypoxia are the factors contributing to perinatal mortality [13].

The ISSHP 2018 has clearly indicated the lack of early pregnancy tests or instruments to predict the onset of pre-eclampsia; however, it does advise patients with a combination of risk indicators, such as high blood pressure, PIGF, and uterine artery doppler, to take 150 mg of aspirin to avoid pre-term pre-eclampsia but not term pre-eclampsia. Integrating first and second-trimester uterine artery pulsatility index (PI) has been found to improve predictive accuracy by up to 45% and have a robust negative predictive value of (92-94%) [14]. Patients with pre-eclampsia and eclampsia should receive high-dependency post-partum care for 3-7 days and anti-hypertensives for at least a week before the stoppage. Counseling regarding birth spacing, contraception, and the incorporation of pre-conceptional education is crucial in future management plans. Approximately 20-50% of subsequent conceptions may be complicated with hypertension, whereas 2-6% can develop HELLP syndrome [15]. The unanticipated problems that arise during pregnancy raise the likelihood of post-traumatic stress disorders. All patients should be followed up at 12 weeks post-partum and undergo yearly health assessment for life along with lifestyle modifications.

As a referral center, our hospital receives a huge load of critical patients. This study was undertaken to provide significant insight into obstetric and critical care provision in the periphery and rural areas.

## Materials and methods

Data collection method

A hospital-based prospective comparative observational study was conducted in the Department of Obstetrics and Gynecology, over a period of one year at Bhaikaka University; the confidentiality and privacy of the participants were maintained. Research commenced after obtaining approval from the institutional ethics committee (IEC/BU/141/46/36/2023). Patients fulfilling the inclusion criteria were enrolled in the study. 

Inclusion Criteria

All cases of antepartum, intrapartum, and postpartum eclampsia admitted in the maternity ward, eclampsia room, and ICU were included. 

Exclusion Criteria

Other causes of convulsion during pregnancy like epilepsy, meningitis, cerebrovascular accidents, and patients presenting beyond puerperium. Patients with chronic hypertension (before 20 weeks of gestation), chronic renal disease, connective tissue disorders, and preeclampsia without convulsion were excluded.

The sampling technique employed was purposive. The sample size was calculated using the hypothesis testing method, and based on the formula, where Þ Z = Z value at 90%; confidence intervals = 1.65, the minimum required sample size was calculated as 116. However, 140 patients including 70 cases and 70 controls were enrolled.

Methodology

This study covered hypertensive patients with tonic-clonic convulsions, whether antepartum, intrapartum, or postpartum, regardless of their age, gestational age, parity, or status of booking at admission. Data was collected with the help of a pre-formed case record form. Using the purposive sampling technique, a similar number of control populations were selected after matching basic demography. Data pertaining to history, antenatal care (ANC) availed during pregnancy, records of blood-pressure measurement and medications, type, nature, number of convulsions before admission, and events prior to convulsions were elicited from patients or attendants. Investigations like complete blood counts, liver and renal function tests, coagulation profile, fundus examination, and urine protein were ordered. Obstetric sonography and Doppler scans, where needed, were arranged.

For classification and identification of hypertensive disorders of pregnancy, the ISSHP (2018), ACOG-2019, and FOGSI GPCR guidelines were taken into consideration. Management was done as per existing protocols in the department. Magnesium sulfate was employed as the drug of choice for controlling convulsions unless contraindicated, in which case, levetiracetam was used. Blood pressure was managed by oral nifedipine, labetalol, or injectable labetalol. The following objectives were determined: Incidence of eclampsia at our tertiary teaching institute. Parameters such as demographic profile, sociopsychological background, risk factors, and maternal and perinatal outcomes.

Statistical analysis

Statistical software STATA 14.2 (StataCorp LLC, College Station, Texas, USA) was used for data analysis. Data was entered into a Microsoft Excel spreadsheet, EPI Info version 7 software. Descriptive statistics [Mean (SD), Frequency (%)] was used to depict the baseline profile of the study participants. To compare continuous and categorical variables between cases and controls, an independent sample t-test and a chi-square test were used respectively. A p-value <0.05 was considered statistically significant.

## Results

There were 1003 births in all during the study period. Of them, 70 had tonic-clonic convulsions, indicating a 0.7% presence of eclampsia. Three twin deliveries were also observed in the case and control groups.

Table 1 outlines comparisons in the demographic profile. The median age in both groups was 25 years. The distribution of primigravidae and multigravida was comparable in both groups. With respect to gestational age, the maximum number of patients in both groups were in their 2nd or 3rd trimester. The majority (50%) of patients presented with antepartum eclampsia; 23% had intrapartum, and 27% had postpartum eclampsia. Regarding the booking status, all the patients in the study group were unbooked, referred from various centers like government institutions (57%), and private hospitals (27%), and the remaining came directly from home (15%), while 45% were booked antenatally among the control participants. Most patients belonged to the upper and lower socioeconomic status in both groups. Just 14% of cases and 4% of controls were Muslims, while the majority of study participants i.e. 85.7% of cases and 96% of controls were Hindus.

**Table 1:** Clinico-demographic characteristics among the study and control subjects Statistical test: Descriptive test, two-sample t-test, and chi-square test

Sr. no	Characteristics	Case N (70)	Control N (70)	Total N (140)	P-Value
1	Age(years) mean	23.7±4.2	24.9±4	140	0.959
2	Parity:				0.461
	Primigravida	51 (72.8%)	47 (67.1%)	98	
	Multigravida	19 (27.14%)	23 (32.8%)	42	
3	Timing of convulsions:				
	Antepartum	35 (50%)	0	35	
	Intrapartum	16(22.8%)	0	16	
	Postpartum	19 (27.1%)	0	19	
4	Multiple pregnancies	3 (4.29%)	3 (4.29%)	6	1.0
5	Booking status:				0.001
	Booked	0 (0%)	32 (45%)	32	
	Unbooked	70 (100%)	38 (54%)	108	
6	Referral status:				
	Private setup	19 (27%)	4 (5.7%)	23	
	Government setup	40 (57%)	10 (14.29%)	50	
	Direct from Home	11(15%)	56 (80%)	67	
7	Socioeconomic status:				0.248
	Lower class	20 (28.9%)	22 (31.4%)	42	
	Upper lower	37 (53.6%)	27 (38.5%)	64	
	Lower middle	10 (14.49%)	17 (24.2%)	27	
	Upper middle	2 (2.9%)	4 (5.71%)	6	
8	Religion:				0.08
	Hindu	60 (85.7%)	67 (95.72%)	127	
	Muslim	10 (14%)	3 (4.29%)	13	

Table 2 displays the spectrum of primary risk factors leading to eclampsia in our study. Poor ANC (61.43%), obstetric high risk (38.5%), medical high risk (35%), and severe anemia (35%) were among the leading high-risk factors.

**Table 2:** Comparison of risk factors among the study and control participants Statistical test: Chi-square test; HDP: hypertensive diseases of pregnancy; ANC: antenatal care; CS: cesarean section

Sr. no.	Characteristics of maternal high-risk factors	Case N (70)	Control N (70)	Total (N- 140)	P-value
1	Severe anemia	25 (35%)	1 (1.43%)	26	0.001
2	Previous 1 CS	5 (7.14%)	6 (8.57%)	11	0.75
3	Past history of HDP	4 (5.71%)	0 (0%)	4	0.42
4	Poor ANC	43 (61.43%)	0 (0%)	43	0.001
5	Medical high risk	25 (35.7%)	4 (5.71%)	29	0.001
6	Obstetric high risk	27 (38.5%)	14 (20%)	41	0.016

With reference to Table 3, cesarean section was the main mode of delivery among both cases (69%) and controls (60%), with vaginal delivery being performed in 27% of cases and 40% of controls. Among the study group, 50% of cases show intensive care unit (ICU) admissions, with an average ICU stay of 4 days and 2 days of ventilatory support. The average length of stay in the hospital was noticeably higher among the eclamptic as compared to the non-eclamptic mothers (7.17 days vs. 4.47 days) (P = 0.001). Massive blood transfusion, which is described by the ASA committee on blood management for hemorrhagic shock as replacement of >1 blood volume in 24 hours or >50% of blood volume in four hours, was prescribed to three patients among the cases. Eleven (15.71%) of the cases left against medical advice were due to monetary and other unspecific issues.

**Table 3:** Comparison of maternal outcome among cases and controls Statistical test: Chi-square test, two-sample t-test; LSCS: lower segment cesarean section; DAMA: discharge against medical advice; ICU: intensive care unit

Sr. no	Characteristics	Case N (70)	Control N (70)	Total (140)	P-value
1	Mode of delivery				0.163
	Vaginal	19 (27.1%)	28 (40%)	47	
	Instrumental	2 (2.86%)	0 (0.0%)	2	
	LSCS	48 (68.5%)	42 (60%)	90	
2	Period of gestation				0.001
	Term	38 (54.2%)	60 (85%)	98	
	Pre-term	29 (41.4%)	10 (14%)	39	
	Very pre-term	3 (4.29%)	0 (0.0%)	3	
3	ICU admission	35 (50%)	0	35	0.001
4	ICU stay (days) (mean)	4±2.36	0		
5	Ventilatory (days) (mean)	2.5±1.6	0		
6	Hospital days (mean)	7±3.8	4±1.7		0.001
7	Massive transfusion	3 (4.29%)	0	3	0.08
8	Critical surgical interventions	8 (11.43%)	0	8	<0.05
9	DAMA	11 (15.7%)	0	11	0.001
10	Mortality	1 (1.43%)	0	1	0.316

**Figure 1:**
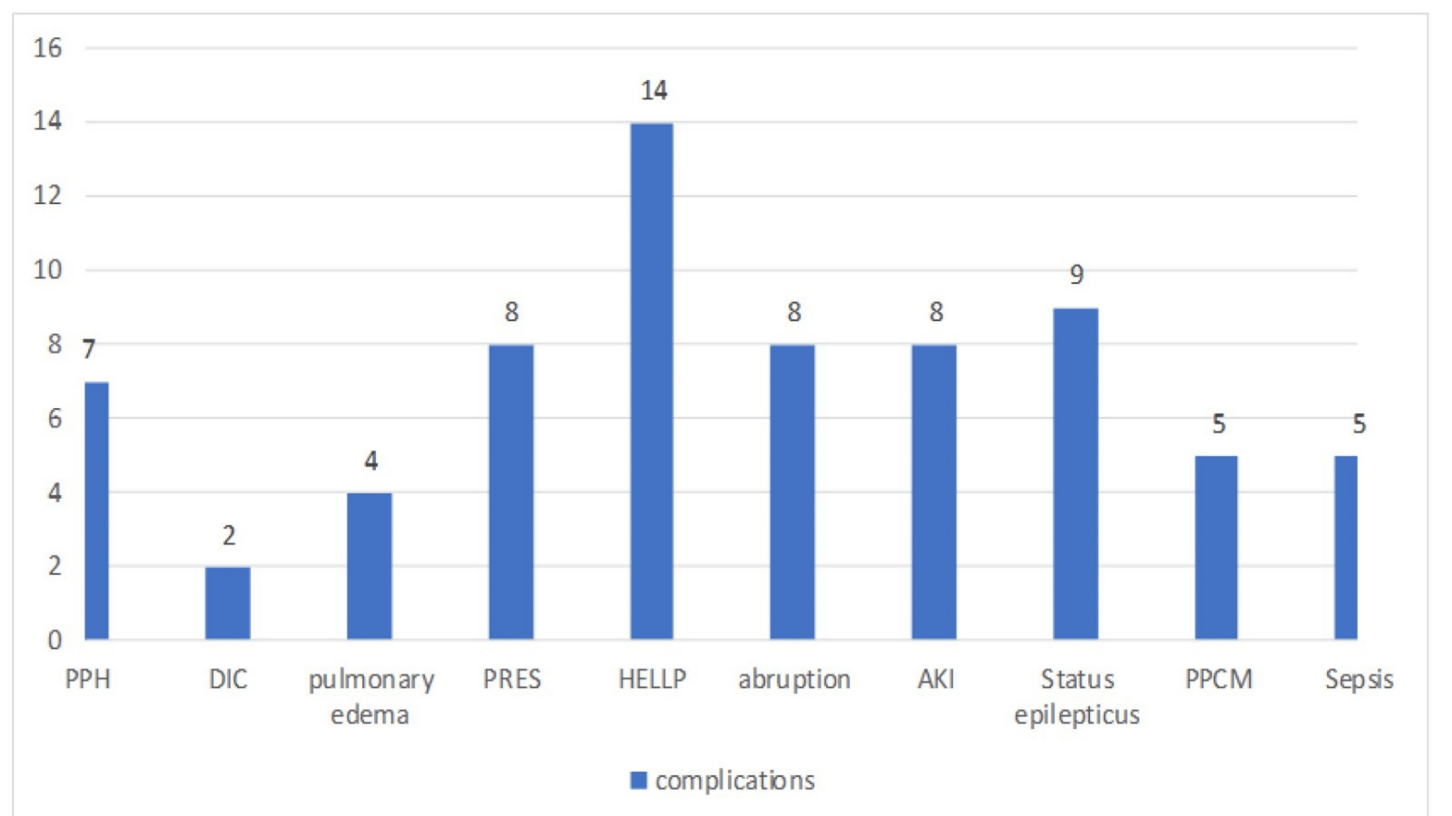
Complications among cases presenting with eclampsia PPH: Post-partum hemorrhage; DIC: disseminated intravascular coagulation; HELLP: hemolysis, elevated liver enzymes, low platelets; AKI: acute kidney injury; PPCM: post-partum cardiomyopathy; PRES: posterior reversible encephalopathy syndrome

Figure 1 displays the complication rates among the cases. More than 20% of cases developed HELLP, while other complications like abruption (11%), acute kidney injury (AKI) (11%), post-partum hemorrhage (PPH) (10%), status eclampticus (11%), posterior reversible encephalopathy syndrome (PRES) (11%), post-partum cardiomyopathy (PPCM) (7%), pulmonary edema (7%), and sepsis (7%) were also frequent.

Biochemical markers like lactate dehydrogenase (LDH), uric acid, and urinary protein were highly deranged among the eclamptic women (Table 4). The majority of the cases (41%) had a single episode of convulsion, while five cases were labeled as status eclampticus. A total of nine patients developed retinopathy; seven of them had grade 1, two had grade 2, and another two had grade 4 retinopathy. All of them had a spontaneous resolution of the disease within a few weeks.

**Table 4:** Distribution of laboratory and clinical outcomes among cases with eclampsia LDH: Lactate dehydrogenase Reference range: LDH- 81-234 U/L; Uric acid- 2.6-6.2 mg/dl; Urinary protein (dipstick urinalysis) = +1 to +4 (30mg/dl to 1000mg/dl); 1+ = 30mg; 2+ = 100mg/dl; 3+ = 300mg/dl; 4+ = 1000mg/dl

Sr. no.	Characteristics	Case N (70)
1	Critical surgical intervention:	
	Obstetrics Hysterectomy	1 (1.43%)
	Cervico-vaginal exploration	4 (5.71%)
	Laparotomy	0
2	Laboratory investigations:	
	LDH (U/L) (mean)	464±588
	Uric acid (mg/dl) (mean)	6.2±1.8
	Urinary protein (dipstick)(mean)	+1.7±1.10
3	Number of convulsions:	
	1	29 (41%)
	2	18 (25.7%)
	3	13 (18.5%)
	4	5 (7.14%)
	5	4 (5.71%)
	>5	1 (1.43%)
4	Grades of retinopathy:	
	0	61 (67%)
	1	7 (10%)
	2	1 (1.43%)
	3	0
	4	1 (1.43%)

Table 5 shows significant differences in perinatal outcomes among the two groups. 10 cases in the study group had non-salvageable babies, while there was no perinatal loss among the control population. The rate of NICU admission among babies of eclamptic mothers was also statistically significant (43.84% vs. 5.48%) (P < 0.001). These newborns had a considerably greater rate of respiratory morbidity (P = 0.005).

**Table 5:** Comparison of perinatal outcome in patients presenting with eclampsia and those without eclampsia Statistical test: Chi-square test; IUFD: intrauterine fetal demise; NND: neonatal death; NICU: neonatal intensive care unit

Sr. no	Characteristics	Case N (73)	Control N (73)	Total	P-value	
				N (146)		
1	Live birth:				0.005	
	Single birth	57 (78%)	67 (91.8%)	124		
	Multiple births	3 (8.2%)	3 (8.2%)	6		
	Total	63 (86.3%)	73 (100%)	136		
2	IUFD	9 (12.3%)	0	9		
3	NND	1 (1.37%)	0	1		
4	NICU admission	32 (43.84%)	4 (5.48%)	36	<0.001	
5	Birth weight				<0.001	
	<1.5 kg	12 (16.4%)	1 (1.37%)	13		
	1.5-2 kg	37 (50.68%)	21 (28.7%)	58		
	>2 kg	24 (32.8%)	51 (69.86%)	75		
6	APGAR Score (mean)	4.2±2.4	7.4±		0.005	

Premature deliveries occurred more frequently in patients with eclampsia. The rate of pre-term births in the study group was significantly higher (41.43%) than in the control group (14.29%), and three of them were very pre-term (4.29%). There were considerably more low-birth-weight babies in the study group than in the control group (P <0.001).

## Discussion

Eclampsia is linked to increased rates of maternal and perinatal morbidity and mortality. Shah et al. studied the uteroplacental bed and noticed several alterations, such as medial necrosis, endothelial damage, intimal cell growth, and the insudation of plasma components into vessel cells. This is believed to result in reduced tissue perfusion, endothelial cell dysfunction, microvascular thrombosis, platelet activation or destruction, and vasospasm [16]. An incomplete invasion of spiral arteries by villous trophoblasts and compounded oxidative stress are thought to result in the induction of systemic microvascular coagulation, increased capillary permeability, and end organ failure [16]. Evidence has been gathered regarding a connection between HLA-DR4 and proteinuric hypertension [17]. Apart from routine blood and urine investigations, special biomarkers and imaging services such as 2D maternal echocardiography, chest X-rays with shields, and neuroimaging may be warranted when there is a clinical suspicion. A clinical predictive model called the PIERS model (Preeclampsia Integrated Estimate of Risk), currently in early research development, is postulated to predict the likelihood of a composite severe unfavorable maternal outcome based on data gathered from 0 to 48 hours following admission with preeclampsia [13].

This study concentrated on a limited case-control population comprising women who experienced eclampsia and those who were normotensive during pregnancy. A mean age of 23 years was witnessed in both the case and control groups. This contrasts with findings from the study by Jantasing and Tanawattanacharoen, which reported an average age of 29 years [18]. Our study thus observed a relatively younger age group compared to the literature. Among the cases, 73% were primigravida, while 27% were multigravida. This distribution corresponds to previous studies in the literature implicating nulliparity as a risk factor. It is also observed that most cases were in the post-partum period, and the majority of them (>80%) required intensive care, highlighting the critical importance of post-partum care provision to mothers.

In the study, it is noteworthy that all the cases were unbooked, a proportion significantly higher than in other studies such as Melese et al., where only 31.9% of cases were unbooked [19]. The lack of early detection of gestational hypertension can be attributed to general ignorance among women and inadequate quality control at rural and semi-urban set-ups offering prenatal care to the rural population. The GESTOSIS score is a useful prognostic tool that frontline healthcare workers can utilize to identify pre-eclamptic women [20].

An efficient referral system plays a crucial role in establishing connections between various levels of care. Developing clear guidelines helps standardize the referral process. Additionally, effective communication and culturally sensitive counseling can enhance patient understanding and acceptance, thus improving compliance and access to appropriate care. Investing time and effort in cultivating relationships with peripheral centers can significantly boost patient care. Given that the majority of patients in the study group were referred from peripheral government hospitals (PHC, CHC, and District Hospital), fostering these relationships becomes even more crucial.

In our study, a past history of hypertensive disease was present in 4% of cases, which can be attributed to the predominance of primigravida patients. This differs notably from other authors, who report rates of 46% in previous pregnancies [21].

Among the study group, 43 patients (61.43%) had inadequate ANC visits during their antenatal period, whereas all patients in the control group had regular ANC visits (P 0.001). This highlights poor ANC attendance as the most common modifiable risk factor among the study group.

In the present study, 27% of cases were delivered vaginally, while 68% required a cesarean section due to various indications, primarily a poor Bishop score. These findings align with those reported by Divyaradha et al., who reported a cesarean delivery rate of 69%, suggesting consistency in delivery methods and indications across different studies [15]. Raghuvanshi et al. (2021) showed an LSCS rate of 70% [22], while Hall et al. depicted an 81.5% cesarean section rate [23].

Eclampsia and HELLP syndrome emerged as the primary causes of maternal ICU admission in the hospital, accounting for 0.6% of all deliveries. This rate is consistent with findings reported by other authors, which typically range from 0.4% to 2.4%. This reinforces the significant contribution of eclampsia and HELLP syndrome to extreme maternal morbidity and the immense economic burden inflicted upon society and families [24]. Among the study participants, around 20% developed HELLP, representing a notably higher incidence compared to the other studies mentioned. As a tertiary care referral center, our setup receives a high load of critical referrals, which could justify the higher incidence of complicated cases.

Certain biochemical markers correlate with the severity of maternal disease and can also predict fetal outcomes. These markers may include assessments of renal function (such as serum creatinine, serum LDH, and uric acid levels), liver function tests (such as serum transaminases and bilirubin levels), and coagulation parameters (such as platelet count and coagulation profiles). Therefore, integrating clinical and biochemical assessments can foster a systematic strategy to manage and optimize outcomes [25]. Minire et al. reported liver damage in 4.9% and renal impairment in 12.3% of cases, while Sindhu found abnormal liver and renal function tests in 19% and 17% of women, respectively [26,27]. The present study documented deranged liver function in 20% and renal function in 11.4% of cases. These findings indicate a significant incidence of end-organ damage in eclamptic mothers, often secondary to late referrals and poor primary management at peripheral health centers. Additionally, a single death was observed among the cases attributed to sepsis and disseminated intravascular coagulation (DIC). Curiel-Bal sera et al. and Quah et al. reported maternal mortality rates of 1.5% and 1.3%, respectively, in their settings [28,29]. This discrepancy, which was seen in comparison to our study, may be attributed to the relatively short duration of our study period. However, Tufnell et al. reported no maternal deaths during a four-year study period in their setting [30].

In a study by Familoni et al., abruption was reported in 6.1% of cases [31]. Conversely, Minire et al. observed abruption in 30 out of 430 subjects, accounting for 6.9% of cases [26]. In our study, abruption was observed in 11.43% of mothers. The number of convulsions is directly correlated with maternal-fetal outcomes. This association may be attributed to hypoxia and metabolic acidosis, which often escalate mortality rates.

The existing literature on seasonal variation presents a diverse range of conclusions. Subramaniam et al. demonstrate a notable rise in the incidence of eclampsia during the monsoon season [32]. However, no seasonal variation was noted at our center.

The neonatal outcome in pregnancies complicated by eclampsia depends on various factors, including the availability of intensive care facilities and the gestational age at birth. Overall, the management of eclampsia requires a delicate balance between addressing maternal health concerns and mitigating the risks to the unborn baby.

The perinatal mortality in the current study was 14.2%. Among these, 12.86% were attributed to intrauterine fetal demise, primarily due to abruption and irreversible hemorrhagic shock to the fetus, often resulting from late referral. These results are in line with research from other regions of the nation where perinatal death rates between 30 and 40 percent have been reported [33]. Neighboring nations like Bangladesh and Nepal have reported similar outcomes [34]. Panaitescu et al. observed a relatively lower incidence of pre-eclampsia in a study in Romania, but higher rates of stillbirths and low-birth-weight infants [35]. The study also noted the presentation of the disease in early gestational periods.

The neonates in these cases were more likely to experience complications. Prematurity and babies born to mothers in a severe condition or who had experienced multiple convulsion episodes were the main causes of NICU admissions. Eclampsia, occurring before the onset of labor (antepartum eclampsia), is strongly associated with preterm birth and compounds the likelihood of neonatal complications like hypoxia and metabolic acidosis.

Appropriate triaging of patients allows for efficient management of eclamptic women in a safe environment. Teamwork is crucial, and adherence to the universal guidelines of airway, breathing, and circulation should be maintained. The baby should be delivered after maternal stabilization. Preventing maternal harm and securing the patient's airway remain the top priorities when they are convulsing or have recently experienced a convulsion. The safe and economical use of magnesium sulfate as an anticonvulsant has been recommended by the WHO. Antihypertensive agents like labetalol, calcium channel blockers, hydralazine, etcetera should be instituted and continued on a case-by-case basis. Labetalol is widely accepted as a first-line and effective antihypertensive. However, it's essential to note that labetalol is contraindicated in certain conditions, including asthma, congestive heart failure, diabetes mellitus, and cases of bradycardia. For patients with eclampsia, these contraindications are essential to take into account when choosing the right antihypertensive medication [20].

Limitations

There was insufficient data regarding early pregnancy because most patients presented in the third trimester and were not scheduled. With a limited sample size, the study was carried out in a single hospital. As such, the results may not be representative of the community as a whole. Information about the long-term outcomes of babies is scarce since the neonatal outcome measures were restricted to the mother's hospital stay duration.

## Conclusions

Maternal mortality and morbidity are important indicators of healthcare services. HELLP, abruption, and multiorgan dysfunction were found to be the most common complications affecting fetal and maternal outcomes in patients with eclampsia. Neonatal asphyxia and preterm birth occurred more frequently among the cases. Most research has been conducted on the Western population. Collaborative research should be encouraged in this direction at population levels for the efficient implementation of safe motherhood programs.
